# Effects of Solution Treatment on the Microstructure and Properties of Al_0.4_Co_0.5_V_0.2_FeNi High-Entropy Alloys

**DOI:** 10.3390/ma19163551

**Published:** 2026-08-21

**Authors:** Hongbo Duan, Wei Yang, Zhijun Ma, Yuan Li

**Affiliations:** School of Materials and Chemical Engineering, Xi’an Technological University, Xi’an 710021, China; xiangongdadhb@163.com (H.D.); xiangongdayx@163.com (W.Y.); xiangongdamzj@163.com (Z.M.)

**Keywords:** high-entropy alloy, solution treatment, spinodal decomposition, mechanical properties, sluggish diffusion

## Abstract

This work explores V-alloyed Al_0.4_Co_0.5_V_0.2_FeNi high-entropy alloys that are solution-treated at 1000–1200 °C for five hours and water-quenched. Solidification of this high-entropy alloy follows the sequence face-centered cubic (FCC) → ordered L1_2_ → body-centered cubic (BCC) → ordered B2. All treated alloys maintain dual FCC–BCC-B2 microstructures, with temperature altering only morphological features. At 1000 and 1100 °C, coherent spinodal decomposition takes place in FCC matrices to form rodlike precipitates with 20.3% lattice misfit; meanwhile, Ostwald ripening lengthens the precipitates from 1.41 to 2.11 μm. Spinodal decomposition is inhibited at 1200 °C, which is accompanied by coarsening and granulation of B2-ordered BCC grains. Slow atomic diffusion prevents recrystallization even above 0.4*T*_m_. Solution heat treatment dissolves the B2 phases to generate supersaturated solutions stronger than the as-cast counterparts. Elevated solution temperatures reduce hardness (243–190 MPa) and tensile strength (919–839 MPa) but boost elongation (26–35%). Fractures display mixed dimple-cleavage features. Overall, a five-hour solution treatment at 1100 °C plus water quenching delivers balanced strength (851 MPa) and ductility (33%), serving as the optimal heat treatment for this alloy.

## 1. Introduction

High-entropy alloys (HEAs) transcend traditional single-component alloy design paradigms by leveraging four core effects: high configurational entropy, lattice distortion, hysteresis diffusion, and cocktail effects, enabling the formation of face-centered cubic (FCC)–body-centered cubic (BCC) composite solution structures [1,2,3,4,5,6]. Since Yeh et al. first systematically proposed the concept of HEAs, AlCoFeNi-based HEAs have become a focal research area in the field of duplex HEAs due to their excellent element compatibility and ease of phase composition control via aluminum and vanadium [7,8,9,10,11]. Appropriate aluminum content promotes the formation of the ordered BCC-B_2_ hard phase, while vanadium regulates lattice mismatch, precipitation behavior, and phase stability through solution doping, achieving a balanced microstructure combining soft FCC phases with hard BCC phases [12,13,14,15]. This multiphase synergy overcomes the traditional bottleneck of strong plasticity inversion in alloys. Studies have demonstrated that AlCoFeNi-based alloys tend to develop dendritic segregation and intergranular network-like secondary phases during solidification, severely compromising overall performance due to compositional inhomogeneity [16,17,18,19]. Solution heat treatment remains the most cost-effective approach to eliminate casting segregation, control precipitate morphology and size, and optimize strength–plasticity performance.

Extensive research has been conducted both domestically and internationally on composition optimization, melting processes, and low-temperature aging of AlCoFeNi-based HEAs. Cao et al. investigated the effects of high-temperature solution treatment on alloy microstructure, demonstrating that heat treatment significantly alters the volume fraction and distribution morphology of the second phase [20]. Other studies have identified amplitude decomposition-induced hierarchical nanoparticle deposition as a key mechanism for achieving dispersion strengthening in FCC-L1_2_ HEAs, with phase morphology determined by the interplay between interfacial energy and lattice mismatch elasticity [21,22,23]. Other studies highlighted that the unique hysteretic effect in HEAs substantially reduces atomic migration rates; even when heat treatment temperatures exceed the theoretical recrystallization temperature (0.4*T*_m_), grain boundary migration and dislocation recovery remain kinetically constrained, preventing recrystallization [24]. However, most investigations have focused on Cr- and Mn-doped systems, while systematic studies on V-modified alloys remain limited. Li et al. employed first-principles calculations to analyze the influence of V content on phase stability, but comprehensive experimental data—including quantitative characterization of precipitates at different solution temperatures, mechanisms of amplitude decomposition, elastic energy calculations, and macro-mechanical coupling—are lacking [25,26]. The evolution pattern of the second phase within the 1000–1200 °C range, along with the intrinsic relationships between continuous hardness reduction, strength decline, and improved plasticity, remains unclear. A complete mechanistic analysis spanning thermodynamic free energy, microscopic coherent interfaces, and macroscopic tensile fracture processes is still lacking.

Dual-phase HEAs possess great application potential. In this paper, the AlCoVFeNi alloy was selected as the research object and subjected to annealing treatments at different temperatures. The effects of annealing temperature on the evolution of FCC–BCC dual phases, lattice characteristics, and grain size of the alloy were investigated, and the correlation mechanism between heat treatment and microstructure was clarified. The microstructure of the alloy was optimized via the regulation of microscale phase constitution and structure, which provides an experimental basis and theoretical support for the precise microstructure design and engineering application of dual-phase alloys.

## 2. Materials and Methods

Al, Co, V, Fe, and Ni, each with a purity of 99.9%, were used as raw materials, which were provided by Beijing Yan Bang New Materials Co. Ltd. (Beijing, China). The experimental HEAs were prepared using the methods of arc melting and copper mold casting (these samples are denoted AC1). The alloys were melted in an argon atmosphere at least five times to ensure the chemical homogeneity of the raw materials in the liquid state, and then solidified in a water-cooled copper mold. Then, solution treatment was conducted in a tubular furnace under an argon atmosphere at temperatures of 1000, 1100, and 1200 °C for 5 h (these samples are denoted AC1000, AC1100, and AC1200, respectively, throughout this study), followed by rapid water quenching.

The phases in the HEAs were identified by a Bruker D8 X-ray diffractometer with Cu Kα radiation (Bruker AXS GmbH, Karlsruhe, Germany), which scanned 2θ between 20° and 100° at a scanning rate of 5°/min in the two-dimensional mode. The collected data were then analyzed using Jade V6.5 software and compared to known powder diffraction files for the phases. The microstructures were characterized using a VEGA IIXMU scanning electron microscope (TESCAN, a.s., Brno, Czech Republic), an optical microscope, and a JEOL JEM-2010 transmission electron microscope (JEOL Ltd., Tokyo, Japan). The chemical compositions were analyzed by energy-dispersive spectroscopy (EDS) (Oxford Instruments, High Wycombe, UK). The specimens used for the TEM analysis were prepared by a precision ion polishing system. For the ion polishing, the specimens were prepared in a disk shape with a 3 mm diameter, and their thicknesses were reduced by grinding to 50–60 μm. Tensile tests to failure were carried out at room temperature and an initial strain rate of 1 × 10^−3^ s^−1^ using an LFM-125 electromechanical machine (Walter+Bai AG, Mägenwil, Switzerland). Thermodynamic calculations of the Al_0.4_Co_0.5_V_0.2_FeNi HEAs were conducted with Pandat software, Version 2022. Vickers microhardness measurements were conducted at an applied load of 200 gf and a dwell time of 15 s. Rockwell hardness testing was performed using a TIME6168 Rockwell hardness tester (Beijing TIME High Technology Ltd., Beijing, China), with a major test force of 150 kgf automatically applied for the Rockwell hardness (HRC) scale and a dwell time of 10 s.

## 3. Results and Analysis

**Figure 1:**
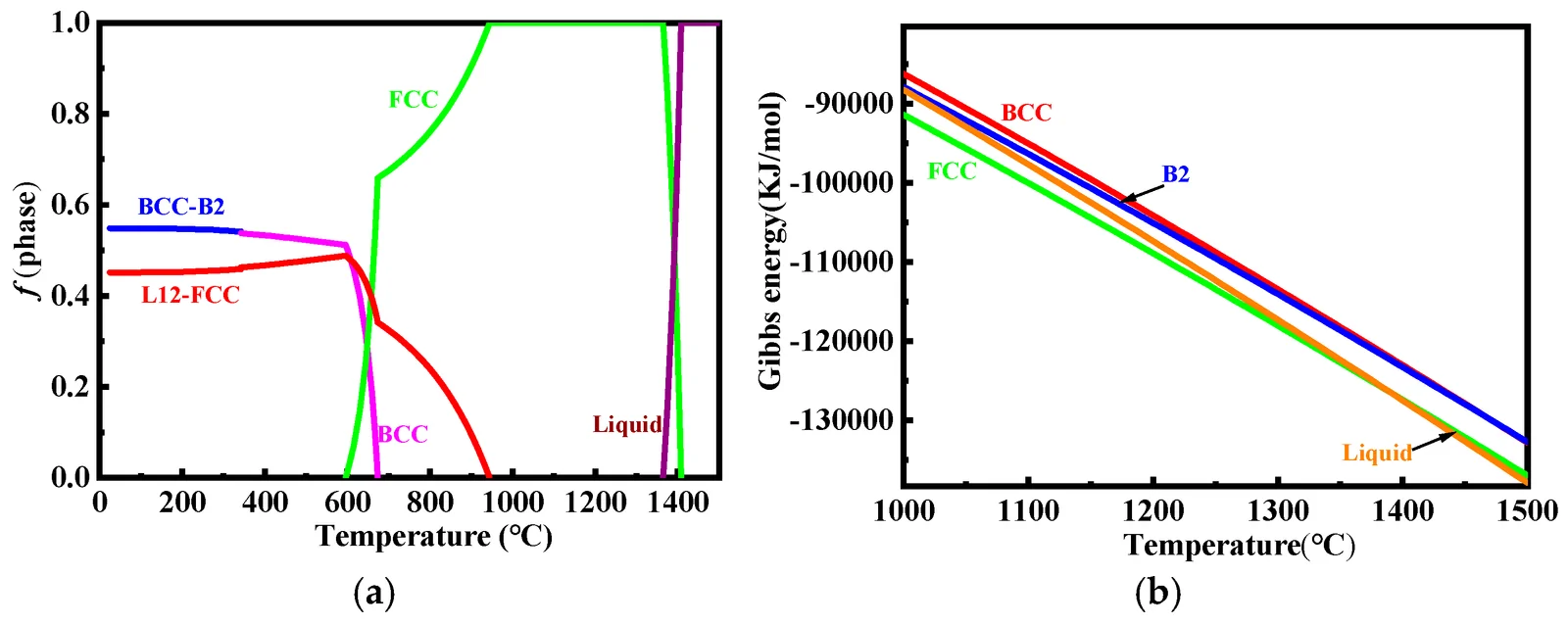
Temperature-phase composition diagram and Gibbs free energy–temperature relationship for the Al_0.4_Co_0.5_V_0.2_FeNi HEAs. (**a**) Temperature-phase composition. (**b**) Temperature–Gibbs free energy relationships.

Figure 1 shows a temperature-phase composition diagram and Gibbs free energy–temperature relationship for the Al_0.4_Co_0.5_V_0.2_FeNi HEAs simulated with Pandat software. As illustrated in Figure 1a, the FCC phase first nucleates in the liquid phase. With decreasing temperature, the FCC-L1_2_ phase begins to precipitate, and its abundance increases with a reduction in the molar fraction of the FCC phase. Further cooling leads to the formation of the BCC phase, which subsequently undergoes a solid-state phase transformation into the BCC-B2 phase. Figure 1b depicts the temperature–Gibbs free energy relationship for the AC1 HEA. It is evident that under equilibrium solidification conditions, when the temperature exceeds 1000 °C, the Gibbs free energy of the FCC phase is at its lowest; solution treatment promotes the interdendritic dissolution of the BCC-B2 phase, thereby enhancing solution strengthening.

**Figure 2:**
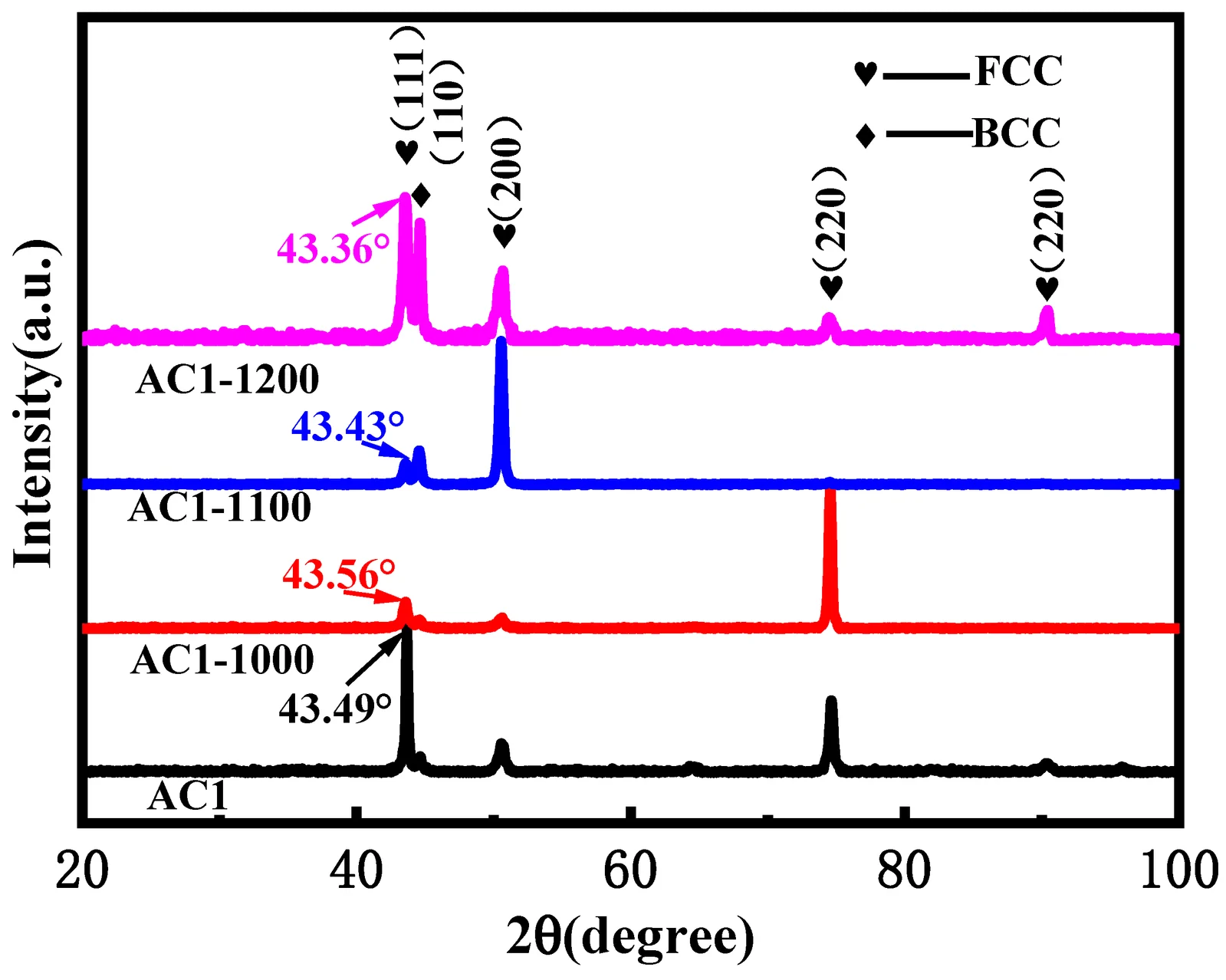
XRD patterns of as-cast and Al_0.4_Co_0.5_V_0.2_FeNi HEAs at different annealing temperatures.

Figure 2 shows X-ray diffraction (XRD) patterns of the as-cast and solution-treated Al_0.4_Co_0.5_V_0.2_FeNi HEAs, which have dual-phase structures composed of FCC and BCC phases. As the annealing temperature increases, the 2*θ* values corresponding to the characteristic main diffraction peaks of the FCC phase shift slightly to lower angles. According to Bragg’s law, 2*d*sin*θ* = *λ*, this shift originates from lattice expansion induced by heat treatment, which leads to an increase in interplanar spacing. The diffraction peaks become narrower and sharper as the annealing temperature rises, indicating the release of residual internal stress and the effective elimination of lattice distortion within the samples. Analysis based on the Scherrer equation, *D* = *Kλβ*cos*θ*, demonstrates that the FWHM(*β*) of the diffraction peaks decreases with increasing annealing temperature, verifying that the average grain size of the alloy gradually increases and exhibits continuous grain coarsening with increasing heat treatment temperature.

**Figure 3:**
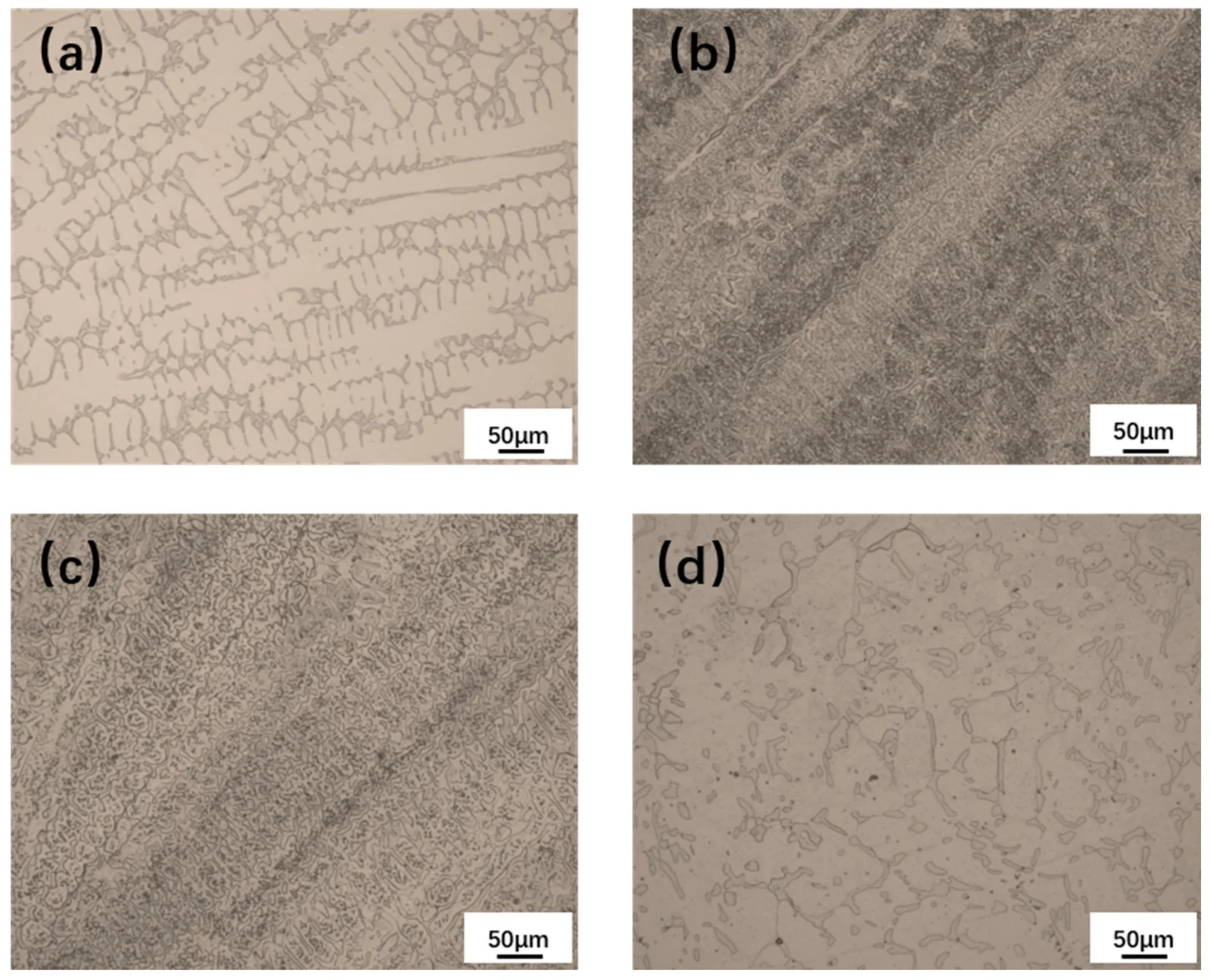
Optical microscopy (OM) photographs of the Al_0.4_Co_0.5_V_0.2_FeNi alloys. (**a**) Cast state Al_0.4_Co_0.5_V_0.2_FeNi alloy. (**b**) AC1-1000 after 1000 °C solution treatment. (**c**) AC1-1100 after 1100 °C solution treatment. (**d**) AC1-1200 after 1200 °C solution treatment.

Figure 3 displays OM microstructures of the different Al_0.4_Co_0.5_V_0.2_FeNi alloys. The results indicate that solution treatment had a minimal effect on the phase composition of the AC1 alloy, which consistently exhibits an FCC–BCC duplex structure; however, it significantly influenced its microstructural morphology. With increasing heat treatment temperature, a second phase with a dispersed distribution forms within the matrices of AC1-1000 and AC1-1100, whereas no such phase is observed in AC1-1200; instead, the second phase between the dendrites demonstrates coarsening and granulation.

**Figure 4:**
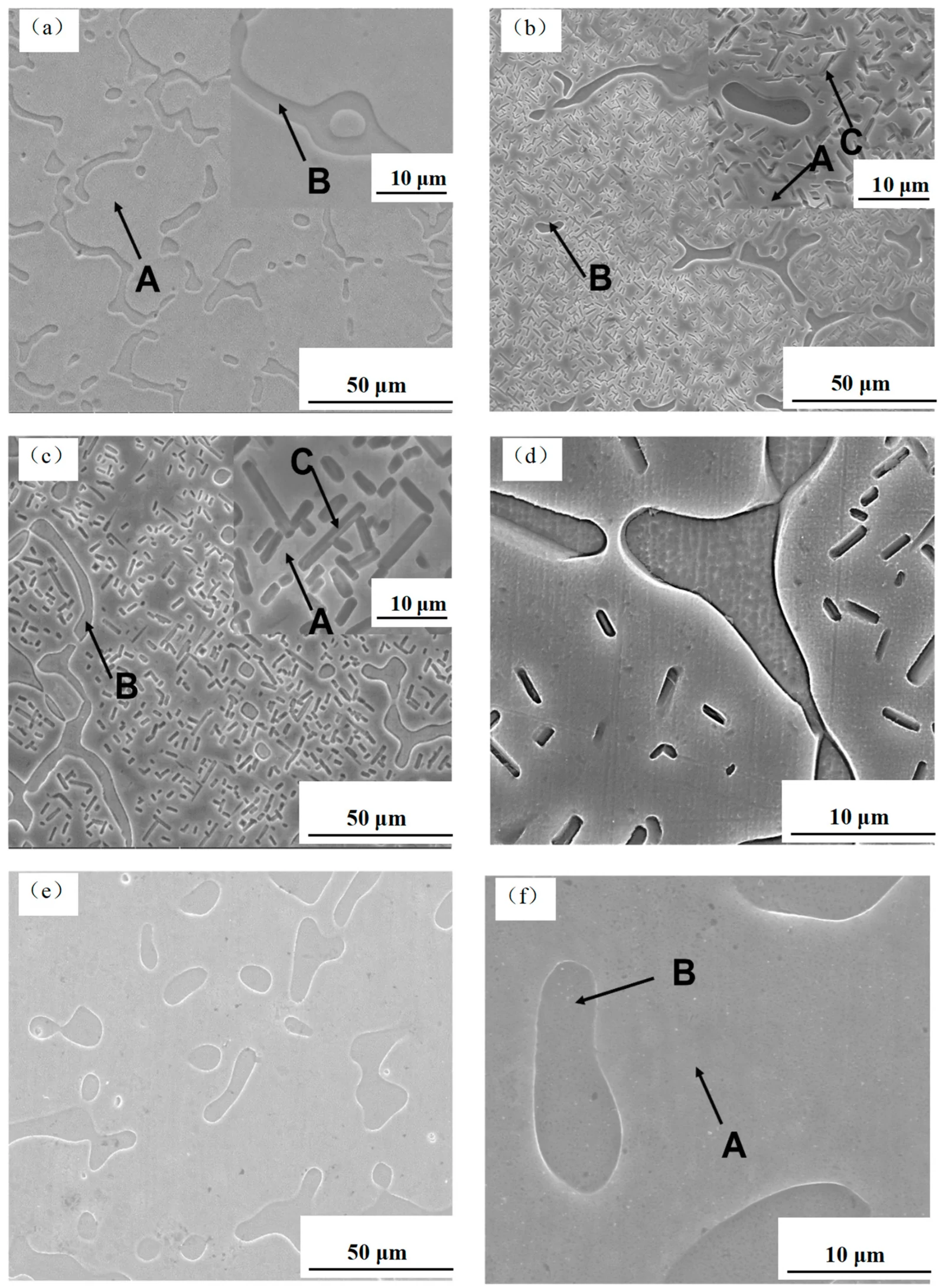
Scanning electron microscopy (SEM) morphology of the as-cast state and solution treatment of the Al_0.4_Co_0.5_V_0.2_FeNi HEA. The phases are marked as A, B, and C, and the compositions of regions A and B are shown in Table 1. (**a**) As-cast Al_0.4_Co_0.5_V_0.2_FeNi alloy. (**b**) AC1-1000 after 1000 °C solution treatment. (**c**,**d**) AC1-1100 after 1100 °C solution treatment. (**e**,**f**) AC1-1200 after 1200 °C solution treatment.

Figure 4 shows SEM images of the microstructures of the Al_0.4_Co_0.5_V_0.2_FeNi HEA, both as-cast and after solution treatment. Table 1 presents the results of an EDS analysis of different micro-regions. Combined with the SEM micrographs, region A is identified as an FCC matrix, region B corresponds to Al-enriched BCC-B2 precipitates, and region C, observed in samples treated at 1000 and 1100 °C, is a transitional microstructure formed during spinodal decomposition. Region A possesses a low Al concentration while being enriched in Fe, Co, and Ni, while region B exhibits obvious Al segregation, verifying that Al aggregation facilitates the formation of the ordered BCC-B2 phase. The Al content in region C lies between that in regions A and B, representing a transitional zone induced by compositional fluctuations during phase transformation.

Table 2 displays the size distribution of precipitates in the HEA matrix at different heat treatment temperatures. Compared to the as-cast morphology, significant changes occur in the two-phase structure after solution treatment: rodlike precipitates form within the FCC phase matrix at 1000 and 1100 °C, with partial solution formation in the BCC phase; at 1200 °C, no rodlike precipitates are observed, but BCC phases between dendrites exhibit island-like structures that gradually granulate and grow. Image analysis using ImagePro software 6.0 revealed that both AC1-1000 and AC1-1100 exhibited short rod-shaped secondary phases in the matrix, with their length increasing from 1.41 to 2.11 μm and diameter expanding from 0.60 to 0.98 μm as the temperature increased from 1000 to 1100 °C. As shown in Figure 1, the alloy’s melting point is approximately 1420 °C, far exceeding its theoretical recrystallization temperature (0.4*T*_m_). However, no recrystallization occurred, which is attributed to inadequate recrystallization kinetics due to multicomponent interactions and hysteresis diffusion effects in the HEAs. These complex elemental interactions hinder dislocation movement and grain boundary migration; under certain conditions, discontinuous phases may form at grain boundaries, further impeding dislocation propagation. Additionally, HEAs exhibit pronounced hysteresis diffusion effects. This means that even when an alloy reaches its theoretical recrystallization temperature, recrystallization does not occur immediately, as the presence of hysteresis diffusion effects complicates the process. In summary, the inadequate recrystallization kinetics caused by the multicomponent nature of HEAs and hysteresis diffusion effects make recrystallization difficult to achieve in this system.

Solubilization is a thermally activated process: the higher the temperature, the faster the atomic diffusion rate. The diffusion process follows the Arrhenius equation, where the relationship between the diffusion coefficient and temperature can be expressed as follows [27]:

(1) $$D=D_{0}exp(-\frac{Q}{RT}),$$

where *D* is the diffusion coefficient, *Q* is the diffusion activation energy, *D*_0_ is a constant, and *T* is the temperature.

Studies have shown that during alloy solidification, the distribution of alloy elements is approximated by the Fourier series components of a cosine function, as illustrated below:

(2) $$C\left(x\right)=\bar{C}+\frac{1}{2}\Delta C_{0}cos\frac{2\pi x}{L}.$$

During solution treatment of dendritic structures, according to Fick’s law and boundary conditions, their concentration varies with time and position at a given temperature:

(3) $$C(x)=\bar{C}+\frac{1}{2}\Delta C_{0}cos\frac{2\pi x}{L}exp\left(\left(-\frac{4\pi^{2}}{L^{2}}Dt\right)\right).$$

The parameter *δ* characterizes the degree of element segregation in the alloy; only the highest and lowest concentration values, namely *x* = 0 and *x* = L2, are considered. Under these conditions, Equation (3) takes the form

(4) $$\delta =\frac{c_{max}-c_{min}}{c_{0max}-c_{0min}}=exp\left(\left(\frac{4\pi^{2}}{L^{2}}Dt\right)\right).$$

In actual solidification, the residual segregation index is generally considered to indicate uniform distribution of the element when *δ* = 0.2. As shown in Figure 4f, the precipitate phase exhibiting BCC dendritic morphology has completely disappeared due to solution treatment; the dendrite spacing is assumed to be infinite. Consequently, near the BCC phase, a higher *L* value corresponds to a greater degree of solution, resulting in localized solution regions around the BCC phase.

**Figure 5:**
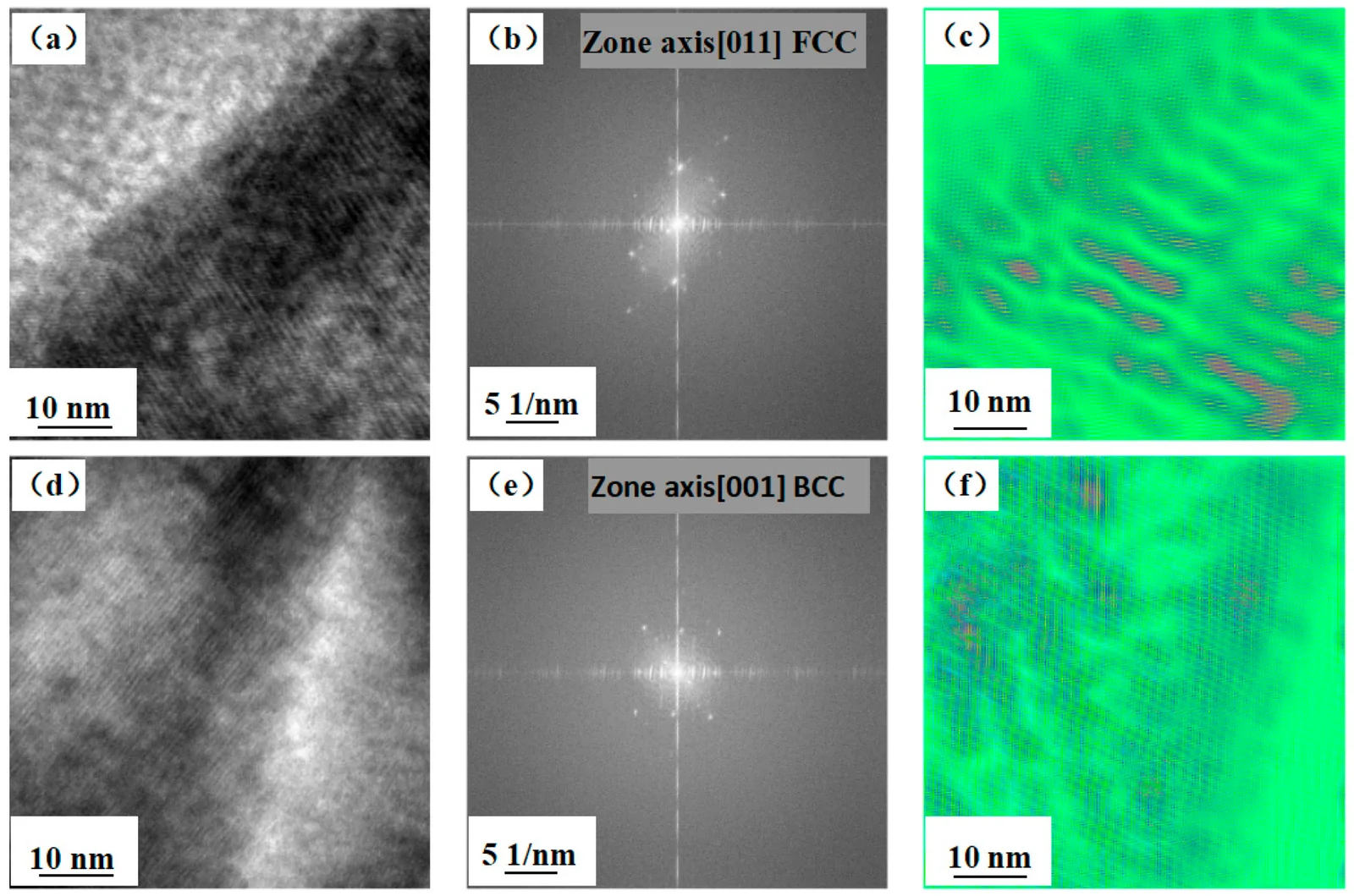
HRTEM morphology and FFT diffraction patterns of the FCC–BCC-B2 phase interface in the Al_0.4_Co_0.5_V_0.2_FeNi alloy. (**a**,**d**) High-resolution morphology of the FCC–BCC phase interface. (**b**) and (**e**) FFT of the FCC-phase matrix. (**c**,**f**) IFFT of the FCC-phase matrix.

Figure 5 shows the high-resolution TEM (HRTEM) morphology, fast Fourier transform (FFT) diffraction pattern, and inverse FFT (IFFT) morphology of the FCC–BCC-B2 phase interface in the AC1 alloy. It reveals that the FCC matrix contains striated and spherical nanoscale precipitates. These precipitates are regularly distributed within the matrix, indicating they are products of amplitude-modulated decomposition. Furthermore, these decomposition products maintain a coherent relationship with the matrix; to minimize their coherent strain energy, they grow along the crystal direction with the lowest coherent strain energy, resulting in a periodic pattern.

The morphology of precipitates is determined by the elastic energy and interfacial energy. For larger precipitates, lattice mismatch between the precipitate phase and the matrix significantly enhances elastic energy. This energy arises from differences in atomic arrangement between the precipitate and the matrix; in AlCoVFeNi alloys, the relative contributions of elastic energy and interfacial energy to the total energy can be quantified using the parameter *L*. In HEAs, increasing precipitate size substantially enhances elastic energy due to lattice mismatch, thereby markedly boosting the overall elastic energy. This increased elastic energy tends to induce precipitate deformation or decomposition to mitigate the energy burden caused by such mismatch, which critically influences precipitate morphology and stability. By adjusting the alloy composition and heat treatment conditions, the precipitate morphology can be optimized for superior performance in specific applications.

The parameter *L* enables precise understanding and control of these energy contributions, facilitating more efficient performance optimization in material design [28]:

*L* = *ε*^2^*C*_44_*r*/*σ*, (5)

where *ε* denotes the lattice mismatch strain (%), defined as *ε* = (*a*_p_ − *a*_m_)/*a*_m_, C_44_ represents the elastic modulus of the matrix (GPa), *r* is the average radius of the precipitate (nm), and *σ* is the interfacial energy between the precipitate and the matrix (J/m^2^).

The precipitates transition from spherical (L = 0) to nearly spherical shapes, branching into a double-symmetric structure when L exceeds 5.6. XRD analysis reveals that the lattice constant of the precipitate phase, α_p_, is 0.286 nm, while that of the matrix phase, α_m_, is 0.359 nm, with a misfit factor, Φ, of 20.3%. First-principles calculations indicate a BCC C4_4_ lattice constant of 108.98 GPa for AC1. Given that AC1’s matrix phase exhibits an FCC structure with a lattice constant of 0.359 nm—close to the Ni lattice radius of 0.352 nm—it can be modeled using the high-temperature alloy Nimonic PE16, which has a Φ value of 0.091 J/m. The equivalent radii are approximately 2 μm for AC1-1000 and 3 μm for AC1-1100. Calculations show L values of 92.7 for AC1-1000 and 137.8 for AC1-1100, demonstrating that precipitate morphology is governed by elastic energy. Numerous studies have reported L values significantly exceeding the critical threshold for rod-shaped precipitates; for example, Ludwig reported an L value of 212 for Cu-rich rodlike precipitates in FeCu alloys [29]. Due to their large size, these precipitates induce lattice mismatch at the interface with the matrix, substantially enhancing the elastic energy and dominating the system’s total energy.

**Figure 6:**
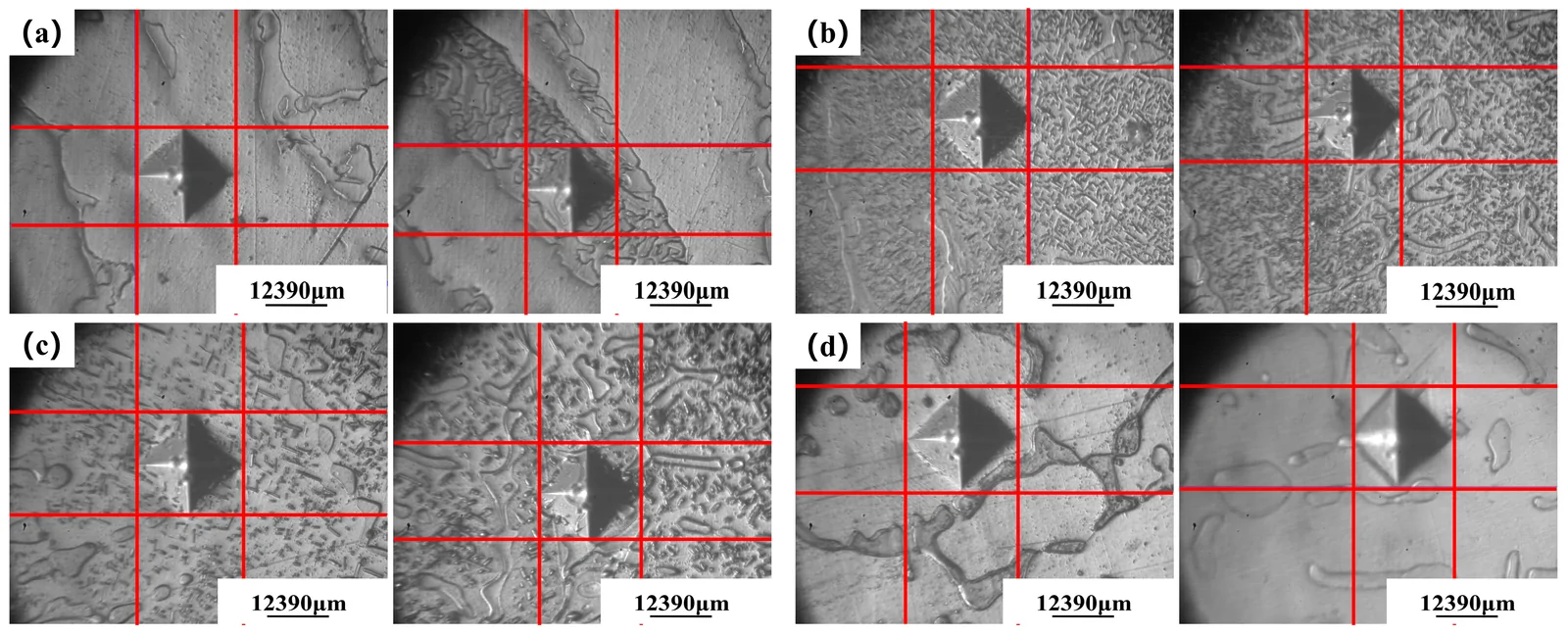
Indentation morphology of Vickers hardness measurements for the as-cast state and solution-treated Al_0.4_Co_0.5_V_0.2_FeNi alloys. (**a**) As-cast Al_0.4_Co_0.5_V_0.2_FeNi alloy. (**b**) Al_0.4_Co_0.5_V_0.2_FeNi alloy after 1000 °C solution treatment. (**c**) Al_0.4_Co_0.5_V_0.2_FeNi alloy after 1100 °C solution treatment. (**d**) Al_0.4_Co_0.5_V_0.2_FeNi alloy after 1200 °C solution treatment.

**Figure 7:**
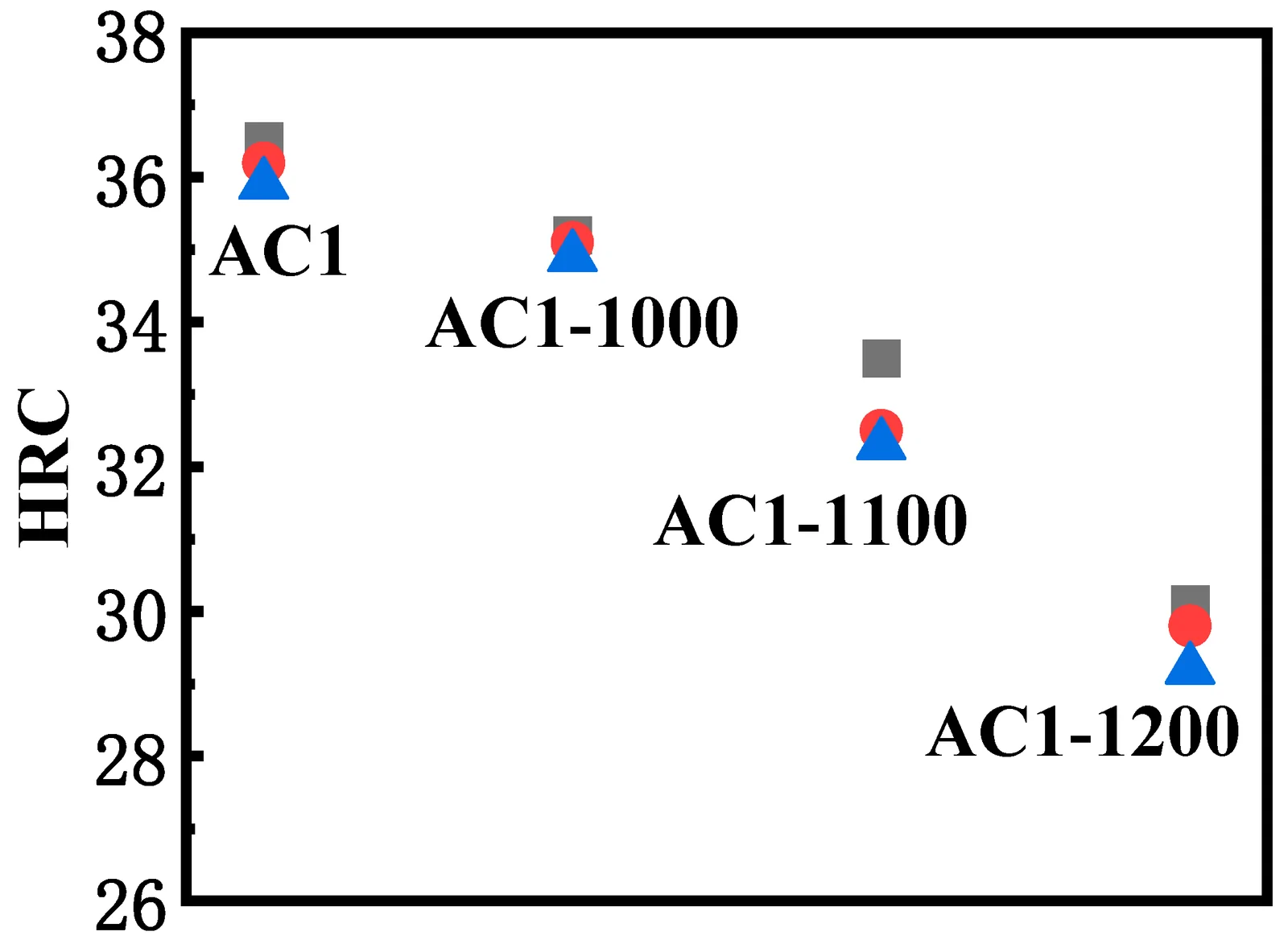
Rockwell hardness measurements for the as-cast state and solution-treated Al_0.4_Co_0.5_V_0.2_FeNi alloys.

Figure 6 shows the indentation morphology obtained from Vickers hardness testing for both the as-cast state and solution-treated AC1 alloy. Figure 7 shows Rockwell hardness measurements for the as-cast state and solution-treated Al_0.4_Co_0.5_V_0.2_FeNi alloys. Table 3 presents the Vickers hardness dimensions for these two states. It is evident that the Vickers hardness decreases progressively with increasing solution treatment temperature, dropping by a hardness value (HV) of 40–50 HV compared to the as-cast state. With an increase in heat treatment temperature from 1000 °C to 1200 °C, the Rockwell hardness (HRC) of the alloy gradually decreased from ~35.1 to ~29.6, which is generally consistent with the Vickers hardness results. Higher solution temperatures enhance intermetallic diffusion, release lattice distortion energy, and promote dendritic recrystallization, resulting in a significant reduction in matrix hardness.

**Figure 8:**
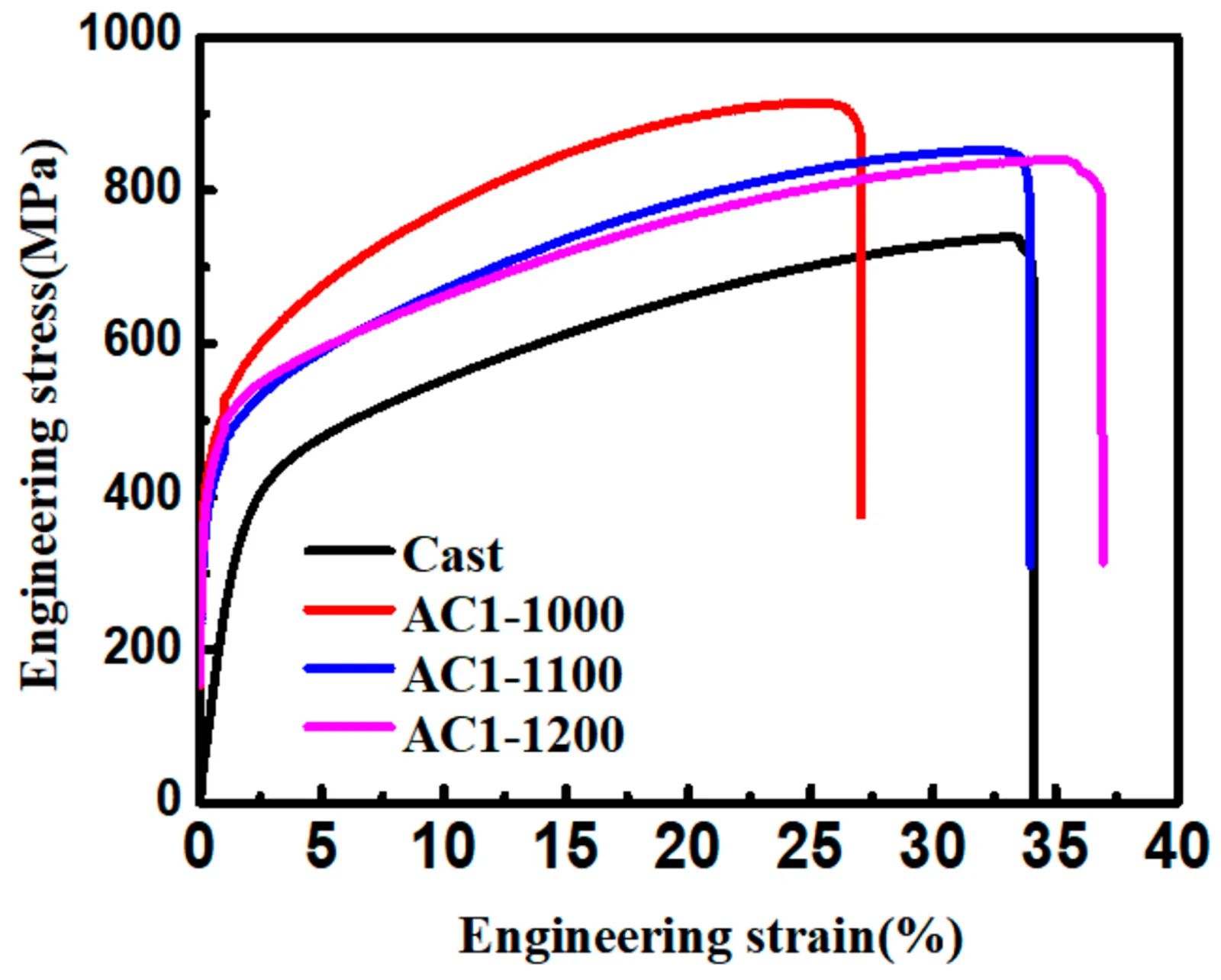
Engineering stress–strain curves of the as-cast and solution heat-treated Al_0.4_Co_0.5_V_0.2_FeNi alloys.

Figure 8 shows engineering stress-strain curves of the Al_0.4_Co_0.5_V_0.2_FeNi alloy in both the as-cast and heat-treated states. It is evident that the strength of the alloy after solution treatment significantly exceeds that in the as-cast state, with strength gradually decreasing as the treatment temperature increases. The tensile strength at 1000 °C is approximately 919 MPa with an elongation of ~26%; at 1100 °C, it reaches ~851 MPa with ~33% elongation; and at 1200 °C, it reaches ~839 MPa with ~35% elongation. These results demonstrate that at treatment temperatures of 1100 and 1200 °C, the alloy’s strength increases by 15.1% and 13.9% compared to the as-cast state, respectively, while maintaining adequate strength and toughness. The alloy treated at 1000 °C achieves the optimal tensile performance under the combined effects of microstructure morphology, precipitate size, and phase fraction. Sufficient spinodal decomposition occurs at 1000 °C, producing high-density, fine, Al-enriched transitional BCC-B2 precipitates in the matrix, which provide the strongest precipitation-strengthening effect. Moderate FCC-to-BCC-B2 phase transformation proceeds at this temperature, generating a relatively high volume fraction of hard BCC-type precipitates that effectively pin dislocations. When the temperature rises to 1100 °C, accelerated atomic diffusion induces coarsening of fine precipitates and weakens precipitation strengthening. At 1200 °C, all transitional phases disappear, partial Al-rich BCC-B2 phases dissolve, and the microstructure coarsens further. Thus, solution treatment can effectively enhance material properties. Under conditions requiring sufficient plasticity, a treatment process involving treatment at 1100 °C followed by five hours of water quenching significantly improves material strength.

**Figure 9:**
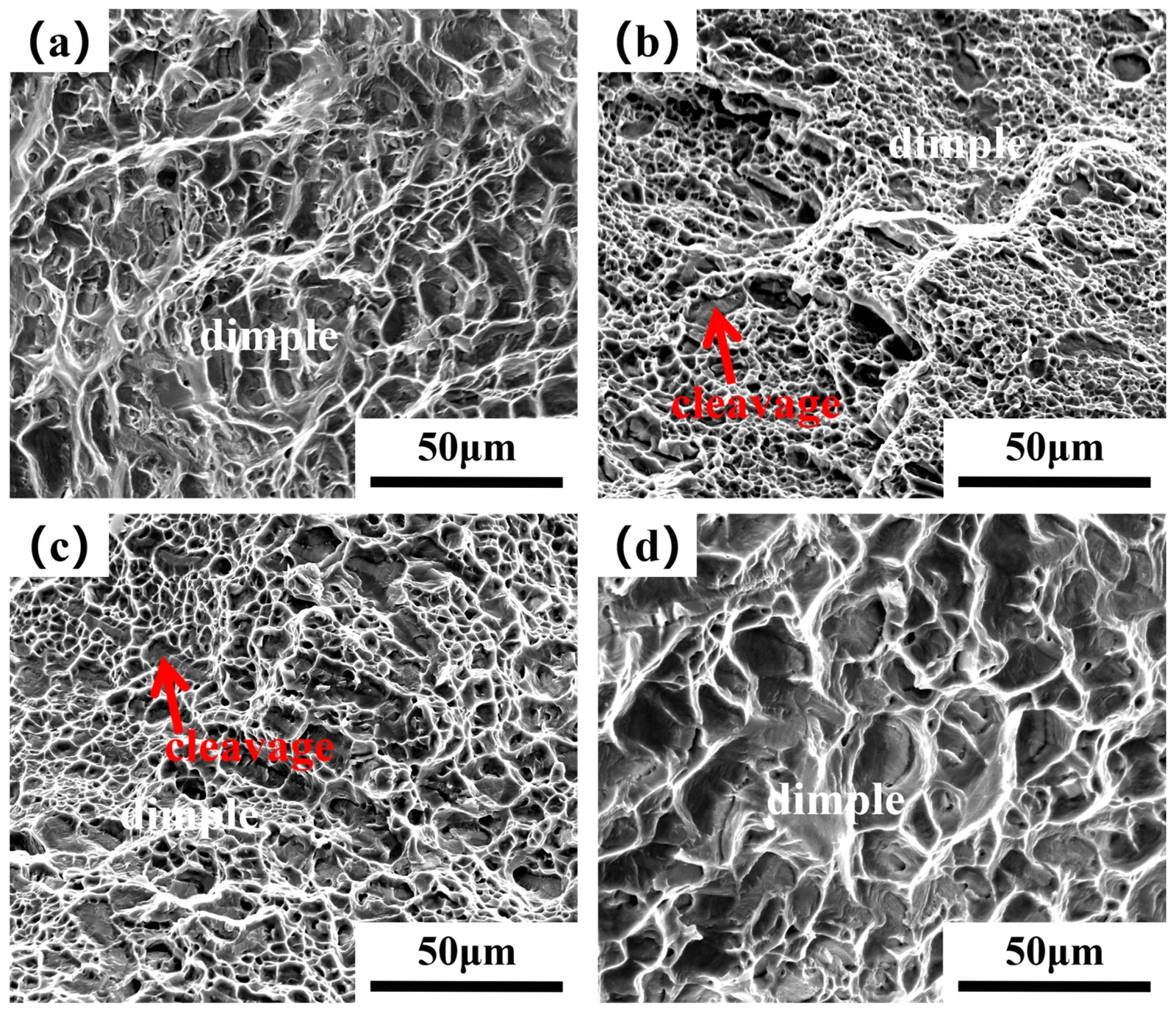
Microstructure of the tensile fracture surfaces of the Al_0.4_Co_0.5_V_0.2_FeNi alloy in both the as-cast and heat-treated states. Tension fracture morphology (**a**) As-cast Al_0.4_Co_0.5_V_0.2_FeNi alloy. (**b**) Al_0.4_Co_0.5_V_0.2_FeNi alloy after 1000 °C solution treatment. (**c**) Al_0.4_Co_0.5_V_0.2_FeNi alloy after 1100 °C solution treatment. (**d**) Al_0.4_Co_0.5_V_0.2_FeNi alloy after 1200 °C solution treatment.

Figure 9 shows the tensile fracture surfaces of the Al_0.4_Co_0.5_V_0.2_FeNi HEAs as-cast and after solution treatment. It is evident that all alloys exhibit both ductile fracture and cleavage fracture: the ductile pits are typical features resulting from tensile deformation of the softer FCC phase in the matrix, while the cleavage steps arise from brittle cleavage fracture induced by stress on the BCC-B2 phase during stretching. Compared to AC1-1200, AC1-1000 and AC1-1100 exhibit numerous dispersed ductile pits and a limited number of cleavage steps; the pit sizes range from 10 to 15 μm, with ductile fracture being the predominant mode. The ductile pits in AC1-1200 are circular or elliptical, exhibiting varying depths and significant size variations, indicating excellent material plasticity.

## 4. Conclusions

The main conclusions of this study are summarized as follows.

(1) The solution temperature solely governs the microstructural configuration. The phase diagrams and Gibbs free energy data indicate that alloy solidification follows the phase transformation sequence FCC → FCC-L1_2_ → BCC → BCC-B2, with FCC exhibiting optimal thermodynamic stability above 1000 °C. After quenching at each temperature, the material consistently maintains an FCC–BCC-B2 two-phase structure; heat treatment cannot alter the equilibrium phases.

(2) At 1000 and 1100 °C, rodlike precipitation in the matrix originates from coherent amplitude-modulated decomposition within the FCC matrix, with the precipitates maintaining a coherent interface with the matrix; calculations using the morphology factor *L* reveal that the *L* value of the precipitates is significantly higher than the critical transition value, indicating that elastic energy due to lattice mismatch replaces interfacial energy as the primary morphology-controlling factor, while increased temperature induces Oswald maturation, leading to substantial size growth of the precipitate phase. At 1200 °C, thermodynamic conditions suppress matrix amplitude-modulated precipitation, causing the original intergranular BCC-B2 phase to coarsen and granulate; multicomponent hysteretic diffusion effects substantially inhibit dislocation and grain boundary migration, and insufficient system kinetics prevent recrystallization throughout the heat treatment process.

(3) In the as-cast state, macroscopic element segregation and enrichment of a hard, network-like BCC-B2 structure induce significant lattice distortion and precipitation strengthening, resulting in the highest hardness in both the matrix and the second phase. Solution heat treatment gradually eliminates solidification segregation and releases lattice distortion through atomic diffusion; however, low-temperature dispersed precipitates progressively recrystallize and coarsen as the temperature increases, progressively weakening the strengthening effect.

(4) Solution treatment involves the dissolution of the BCC-B2 phase into the matrix to form a supersaturated solution, resulting in overall strength that is superior to that of the as-cast state. At 1000 °C, fine rodlike precipitates contribute to solution strengthening, achieving peak tensile strength; however, dense precipitation can induce stress concentration and limit plasticity. As the temperature increases, precipitates coarsen and redissolve, leading to decreased saturation of the solution and a weakened strengthening effect, which gradually reduces the strength, while reduced dislocation slip resistance enhances plasticity continuously. All fracture surfaces exhibit a combination of dimple and cleavage fracture modes. Heat treatment at 1100 °C achieves an optimal balance between strength and plasticity, representing the most favorable process for this alloy’s comprehensive performance.

## Figures and Tables

**Table 1:** EDS results for the as-cast and the solution-treated Al_0.4_Co_0.5_V_0.2_FeNi HEAs.

Element	AC1		AC1-1000			AC1-1100			AC1-1200	
	A	B	A	B	C	A	B	C	A	B
Al	7.83	18.27	8.73	23.25	13.95	8.82	22.45	10.18	10.37	21.22
V	6.84	5.77	7.61	4.2	6.6	6.79	4.71	6.46	6.75	4.87
Fe	36.22	24.27	35.2	21.33	32.15	35.22	22.3	32.6	34.0	23.73
Co	17.60	15.07	17.99	15.25	17.06	18.68	15.6	17.83	18.55	15.58
Ni	31.52	36.61	30.47	35.97	30.24	30.49	34.94	32.92	30.33	34.6

**Table 2:** Precipitate phases in the heat-treated Al_0.4_Co_0.5_V_0.2_FeNi HEA matrix.

Heat Treatment Process	Area/μm^2^	Perimeter/μm	Size (Length)/μm	Size (Width)/μm
AC	0.66	3.90	1.41	0.60
Water quenching at 1100 °C for 5 h	1.41	6.09	2.11	0.98

**Table 3:** Vickers hardness indentation dimensions and HV of Al_0.4_Co_0.5_V_0.2_FeNi alloy in the as-cast state and after solution treatment.

Sample State	Matrix (μm × μm)	Hardness	Dendritic (μm × μm)	Hardness
AC1	39.88 × 38.23	243	36.11 × 36.34	282
AC1-1000	40.12 × 38.94	201	37.05 × 37.52	233
AC1-1100	42.48 × 40.59	197	41.30 × 38.71	232
AC1-1200	43.89 × 42.24	190	39.89 × 39.41	236

## Data Availability

The original contributions presented in the study are included in the article, further inquiries can be directed to the corresponding author.
